# Supplementary material for: Are carbon emissions trading and green financial instruments synergistic? -Comprehensive quantitative research based on content analysis
Source: PLoS One. 2024 Mar 7;19(3):e0298601. doi: 10.1371/journal.pone.0298601 (PMC10919601; doi:10.1371/journal.pone.0298601)
Supplement: S1 Data — (ZIP) [file pone.0298601.s001.zip › Data/地级市Z_F工作报告.zip!/╣π╬≈/╖└│╟╕█╩╨/└┤╫╘╣1⁄2╓┌║┼í╛┬φ┐╦╩2╛▌═°í┐_2015.docx]

各位代表：

现在，我代表市人民政府，向大会作政府工作报告，请予审议，并请市政协各位委员和其他列席人员提出意见。

2014年工作回顾

过去一年，面对国际经济环境复杂多变、国内经济下行压力加大、两次超强台风袭击等严峻形势，全市各族人民在自治区党委、政府和市委的坚强领导下，坚持“稳中求进、改革创新”总基调，以“三年两活动”为抓手，攻坚克难、主动作为，统筹做好稳增长、优结构、增效益、惠民生等各项工作，全市经济社会保持良好发展态势。

一年来，我们主要抓好以下工作：

（一）着力稳增长调结构，经济保持稳中有进

针对国内经济下行的严峻形势，我们认真贯彻中央、自治区稳增长决策部署，采取有力措施，推动全市经济实现平稳较快增长。

多项指标位居全区前列。全年实现地区生产总值588.9亿元，增长10.4%，增速居全区第2，人均GDP突破1万美元大关；规上工业总产值1138.2亿元，增长18%，增速居全区第2；财政收入65.3亿元，增长10.2%；港口吞吐量1.15亿吨，增长8.9%；外贸进出口总额54.7亿美元，增长27.3%，总量居全区第2。人均GDP、财政收入、农民人均纯收入等13项指标总量或增速居全区前列!

工业大幅增量扩能。以龙头企业为支撑，以成长型中小企业为重点，强化企业服务，不断壮大企业规模，全年新投产规上工业企业4家，实现规上工业增加值288.5亿元，增长18%。产值超亿元企业达125家，超10亿元企业18家，超100亿元企业2家，超200亿元企业1家。

农业生产稳步增长。面对“威马逊”、“海鸥”两次历史罕见的超强台风，采取非常措施，多方筹措资金10多亿元，迅速开展生产自救和灾后重建工作，推动农业稳定发展，全年实现农业总产值118亿元，增长3.9%。规划建设4个现代特色农业示范区和56个“一村一品”样板村屯，东兴现代特色海洋渔业核心示范区通过自治区验收。上思澳洲坚果基地等5家农业产业基地被评为国家级农业标准园。万亩“双高”糖料蔗基地、现代蔬菜示范基地、石斛产业基地、水产畜禽标准化养殖示范基地等项目加快建设。新增自治区级龙头企业2家，新成立农民专业合作社57家，发展家庭农场43家。

第三产业持续向好。旅游业加快发展，全年接待国内外游客1183万人次，旅游收入80亿元，分别增长21%、23%。西湾景区成功创建4A级景区，全市A级景区达12个，乡村旅游区（点）34个，星级酒店29家。东兴获评广西首批特色旅游名县。商贸物流保持快速增长，社会消费品零售总额91.7亿元，增长12.6%。全市物流企业超400家，东湾、冲仑物流园总产值超350亿元。房地产持续发展，商品房销售面积156万平方米，增长7.1%。金融服务能力增强，引进2家全国性货币兑换机构，桂林银行防城港分行挂牌成立。设立首期10亿元北部湾（防城港）产业投资基金，成为广西首支地市级产业基金。率先在全区出台扶持企业“新三板”上市办法。全市小额贷款公司数量居全区前列。

（二）奋力上项目强基础，发展后劲持续增强

开展项目建设落实年活动，实施1020个总投资6824亿元的“十百千”项目建设计划，完成固定资产投资478亿元，不断夯实发展基础。

项目建设实现大提速。以六大产业为重点，全力推进项目建设。全年新开工项目323个，竣工投产164个，续建161个，开展前期工作215个，招商引资157个。金川一期、澳加粮油等一批项目建成投产；钢铁冷轧、中电二期、中海油LNG、科元、海森特等一批项目全面开工；核电一期、盛隆二期、信润石化等一批项目加快建设；核电二期、龙成、钧富凰、冠亚等一批项目前期工作快速推进。

重大基础设施建设迈出新步伐。抓好港航基础设施建设，建成402号泊位、武钢20万吨级铁矿石和5万吨级废钢进口专用码头；513-516号泊位、马鞍岭1、2号旅游码头等项目加快建设。加快构建立体交通枢纽，针鱼岭大桥等“双二十”项目基本完成，开工建设防城至江山一级公路、马路至峒中公路扩建、江山半岛环岛东路、龙门跨海大桥西引道、北仑河二桥等一批交通项目；加快推进企沙至茅岭一级公路、钦防高速公路扩建和防城至东兴铁路、铁路云约站、支线机场等项目前期工作。加强城市基础设施建设，文昌大道东段、东湾大道、西湾环海大道和市中心区、城南新区、常山新区、桃花湾片区等路网建设加快，220千伏海潭线、500千伏核海线II回路等输电线路投入运营。城市建成区不断扩大，城镇化率达54%，居全区前列。

项目保障取得新成效。重点破解管理、征地和融资等项目推进难题。创新项目审批机制，实施串联改并联、前置改后审，项目落地速度大幅加快；推行连片征地和项目征地，完成征地3万多亩，其中港口、防城两城区分别完成征地1.23万亩、1.27万亩，全市征地面积超过前2年之和。盘活国有建设用地1.8万亩，保障了624个项目用地，项目用地用海保障率达89%。拓宽融资渠道，成功申报发行企业债券16亿元，全年用于项目建设支出的财政性资金82.7亿元。

（三）强力抓改革促开放，先行先试实现重大突破

充分利用东兴试验区平台，努力将国家战略聚焦优势转化为发展优势，敢闯敢试，改革开放步伐加快。

先行先试创多项全国全区第一。创造了全国第一批进境粮食指定口岸、第一个人民币与越南盾特许兑换业务试点、第一个东盟货币服务平台、第一家跨境保险服务中心等四个“全国第一”，创造了广西第一个启动城乡居民医保统筹改革试点、第一个启动企业登记制度改革试点、成功办理第一笔跨境人民币贷款业务等三个“广西第一”。全年新登记市场企业主体2414户，增长50%。个人跨境贸易人民币结算总量达193亿元，增长1.8倍。边境贸易人民币结算占比达98%。东盟货币业务中心累计交易40亿元。

跨境经济合作区建设加快。按照建设国家第一批跨境经济合作区的要求，不等不靠，制定跨合区建设方案，完善总体规划编制，统筹谋划围网区、配套区10＋70平方公里范围内的产业布局。2平方公里启动区进入场地平整，“一门一桥一厂一馆六路”加快建设，启动配套区标准厂房建设，一批企业已入驻。

开放合作水平全面提升。全年外贸进口额39.6亿美元，增长23%，保持全区第一，出口额15.1亿美元，增长39.6%，获自治区对外贸易进步奖；边贸成交额273.5亿元，增长13.3%。成功举办“魅力东盟·走入中国”文化之旅、东兴试验区专场推介会、东兴—芒街商贸旅游博览会等一批商贸文化交流活动，签约项目47项，项目总投资超500亿元。企业“走出去”步伐加快，在东盟各国设立办事处的我市企业数量居全区首位。

（四）致力办实事惠民生，社会事业持续进步

扎实做好为民办实事工作。全年民生支出73亿元，增长25%，比公共财政预算支出高15个百分点。年初确定的131个民生项目全部完成，累计完成投资28.4亿元。政府承诺为民办10件实事，件件有落实。

“三大攻坚”成效初显。教育攻坚实施八大工程，投入3.1亿元，开展中小学校舍维修和农村义务教育薄弱改造工程，建成市第四中学以及中心区苗壮幼儿园、9所乡镇中心幼儿园；开工建设市理工职业学校新校区、市高级中学二期扩建、市五中、市六中及文昌小学、珍珠小学等一批项目。卫生攻坚实施五大工程，建成市第五人民医院、港口区人民医院新院，开工建设市第一人民医院儿科楼等一批项目。实施十万大山扶贫攻坚计划，投资6.41亿元，完成项目163项，全市脱贫1.4万人。

城乡居民收入持续增长。城镇居民人均可支配收入26523元，增长8.6%；农民人均纯收入9524元，增长11.3%，两项指标总量保持全区前列。贫困村人均纯收入6152元，增长13%。城市与农村低保月人均补助分别提高到245元、110元。

社会保障水平不断提高。城乡居民养老保险和基本医疗保险覆盖率达92%。新增城镇就业2.38万人，转移农村劳动力1.86万人，城镇登记失业率1.78%。争取国开行棚户区改造贷款30亿元，开工改造棚户区12152套，建成保障性住房2230套，完成农村危房改造4000户。新建农村饮水安全工程80处，解决2.8万人安全饮水问题。全市行政村通村道路100%实现硬化。

其他各项社会事业稳步发展。创新驱动能力进一步增强，启动云朗科技园建设，新增自治区级创新型企业1家，新建产学研合作基地3个，首次承担国家863计划子课题。每万人发明专利拥有量增长2倍，上升至全区第5位。上思县列入国家知识产权强县工程试点。文化建设有新成效，国内首部京族题材大型音乐剧《过桥风吹》完成首演，《国旗街的故事》、《金花灿烂》等一批节目获自治区奖项。建成30个村级公共服务中心，江平文化站成为首批全国10个优秀基层文化站之一。首次承办自治区柔道和举重比赛，获国内外各类比赛33金27银12铜。深入开展“美丽家园·清洁乡村”活动，实施“村收镇运县处理”试点，推进59个垃圾综合处理示范村建设，全市卫生环境进一步改善，成功获评“自治区卫生城市”称号。城市环境空气质量优良率达98.6%，优良天数达360天，主要污染物排放量控制在自治区要求范围内。防城区入选全国生态文明示范工程试点区。完成江山乡等4个乡撤乡设镇工作。单独两孩政策稳妥实施。质量技术监督和食品药品安全监管不断加强。我市连续十年被评为自治区安全生产优秀单位。高度重视信访工作，矛盾纠纷排查调处工作取得新成效。“六五”普法教育活动深入开展。强化边海防管控，开展反走私、反恐、缉毒禁毒等专项行动，严厉打击违法犯罪活动，保持民族团结、社会和谐稳定。

一年来，我们始终把民生工作放在首位，全面发展社会事业，让人民群众享受到更多改革发展成果，把“福”字写得越来越大！

（五）大力转作风提效能，政府建设不断强化

扎实开展党的群众路线教育实践活动，着力抓好作风建设，强化依法行政，不断提升政府执行力。

工作作风明显转变。针对“四风”存在的突出问题，狠抓整改落实，着重解决港口粉尘污染等一批群众普遍关注的难题。认真落实中央八项规定，市政府印发文件压缩29%，开展清退干部职工违规多占住房等专项整治，“三公”经费下降11.4%。深化行政审批制度改革，项目审批从平均450天缩短为160天，窗口即时办结和当日办结审批事项占比超过30%。在广西率先开展工商登记“先照后证”、“三证合一”改革试点。开展民主评议科长活动，加强庸懒散问题治理。企业对我市政务环境满意度达81%，跃居全区第四。

依法行政不断加强。制订《防城港市政府重大行政决策程序规定》。率先在全区开展政府部门权力清单制度建设，抓好清权确权晒权制权，做法和成效得到《人民日报》等主流媒体的关注宣传。自觉接受人大法律监督和工作监督，主动接受政协民主监督，人大代表建议和政协委员提案办复率100%。进一步密切与各民主党派、工商联、无党派和社会人士联系。

一年来，统计、审计、国资、司法、税务、海关、检验检疫、海事、通信、邮政、供销、粮食、物价、地震、气象、人防、消防、应急、保密、档案、广播电视、新闻出版、机关事务、决策咨询、社会科学、地方志、民族宗教、残疾人、红十字、妇女儿童等工作取得新进步，双拥共建、国防动员和后备力量建设进一步加强。

各位代表！当罕见超强台风“威马逊”来临时，我们全市动员，精密部署，众志成城，避免了重大人员伤亡。台风过后，面对满目疮痍的家园，全市上下不等不靠，党政军警民齐上阵，迅速重建，恢复生产，使这场特大自然灾害损失减少到最低程度。我们成功抵御超强台风，体现出防城港市人民积极向上、不怕困难、敢于胜利的大无畏气概！体现出防城港市人团结一心、刻苦耐劳、坚韧不拔的精神风貌！抗台救灾取得的巨大成绩，必将激励防城港市人满怀信心、战胜困难、奋力前行！

各位代表！过去一年，我市在艰难形势下经济社会保持平稳较快发展，成绩来之不易。这是自治区党委、政府和市委正确领导的结果，是市人大、市政协有效监督、大力支持的结果，是中直、区直单位和驻军驻警支持帮助的结果，是各级各部门和全市人民团结拼搏的结果。在此，我谨代表市人民政府，向全市广大干部群众，向解放军指战员、武警官兵和公安民警，向各民主党派、工商联、无党派人士、人民团体，向关心支持我市发展建设的各界人士和海内外朋友，表示衷心的感谢和崇高的敬意！

在肯定成绩的同时，我们也清醒地看到，我市经济社会发展仍然面临较多困难和问题。主要是：经济下行压力加大，投资拉动不足；节能减排困难多，转型发展任务繁重；城市配套功能不够完善，服务业发展滞后；政府性债务较重，“小马拉大车”格局没有改变；政府职能转变亟待加大力度，各项改革措施有待进一步落地。针对这些困难和问题，我们一定高度重视，采取有力措施认真加以克服和解决。

2015年工作安排

今年既是全面深化改革的关键之年，也是全面完成“十二五”规划的收官之年。新的一年，世界经济不确定因素依然存在，国内经济发展速度、方式、结构、动力正在发生重大转变。做好今年的工作，必须主动适应经济发展新常态，把握我市机遇叠加优势，突出抓好项目建设，重大项目早建成一天，就多受益一天，投资力度大一些，经济增长后劲就足一些。我们要统一思想，提高认识，以强烈的忧患意识和责任意识，以一抓到底的工作作风，开拓进取，敢做善成。

今年政府工作的总体要求是：全面贯彻党的十八大、十八届三中、四中全会和习近平总书记系列重要讲话精神，按照自治区党委、政府以及市委的决策部署，坚持稳中求进工作总基调，积极抢抓国家建设“一带一路”、自治区实施“双核驱动”等战略机遇，主动适应经济发展新常态，把转方式调结构放在更加重要位置，突出创新驱动，以打造边海经济带为主线，以发展六大经济业态为着力点，全面深化改革，扩大开放合作，切实保障和改善民生，继续保持高于全国、全区的发展速度，全面完成“十二五”规划的各项目标任务。

今年经济社会发展的主要预期目标为：地区生产总值651亿元，增长10%；财政收入70.5亿元，增长8%；全社会固定资产投资525亿元，增长10%；规上工业总产值1320亿元，增长16%；社会消费品零售总额102亿元，增长11%；外贸进出口总额63.5亿美元，增长16%；港口吞吐量1.25亿吨，增长9%；城镇居民人均可支配收入29000元，增长9.2%；农民人均纯收入10570元，增长11%；城镇登记失业率控制在2.5%以内；人口自然增长率控制在10‰以内；万元地区生产总值能耗和主要污染物排放量控制在自治区要求范围内，各项社会事业取得新成效。

围绕以上目标要求，我们将重点做好以下十项工作：

（一）推进边海经济带建设，优化发展空间布局

充分发挥我市沿边沿海优势，谋划布局港口经济、海洋经济、口岸经济、旅游经济、生态经济和互联网经济等六大经济业态，打造边海经济带，形成边海联动、点轴配合、产业集聚的经济发展新格局。

合理布局临海产业。以企沙半岛、渔氵万半岛、江山半岛、京族三岛为重点，以企沙大道、北部湾大道、边海大道为轴线，打造产业集聚新高地。企沙半岛要加快发展临港大工业，以钢铁、有色金属、石化、装备制造、能源产业为支点，延伸产业链，形成以港口为核心的产业集群；渔氵万半岛要依托大港口，发展港口物流、仓储配送、加工贸易、专业市场、金融保险、信息咨询等配套生产性服务业，推动港口经济集聚发展。江山半岛要以创建5A景区为重点，引进一批国际知名连锁品牌酒店，建设一批高端接待设施，推动旅游经济发展；京族三岛要重点推进江平工业园改造升级，推进海产品交易及冷链物流系统建设，打造我市首个海洋经济产业园。以海洋经济为引领，通过优化布局，不断壮大临海产业，努力把我市沿海一带建设成为广西重要的经济增长极。

夯实沿边经济发展基础。以东兴至峒中口岸群为重点，以沿边公路为轴线，加快形成沿边产业集群，带动边境地区经济发展。发挥跨境合作区作为广西沿边开放发展的战略支点优势，抓好启动区和配套区产业规划，打造区域性进出口贸易集散中转基地和进口原材料加工出口基地。利用东兴、峒中口岸和里火、滩散互市点的优惠政策，发展加工贸易、跨境旅游、口岸物流等外向型产业，加快构建跨境产业集群，形成我市新的经济增长点。

推动边海联动发展。谋划“十三五”规划，优化沿海地区发展布局，进一步加大沿边地区发展力度，积极推动边海地区优势互补发展，构建边海联动发展新格局。按照国家《沿边地区开发开放规划》要求，做好与《东兴试验区建设总体规划》衔接，启动沿边开发开放带规划编制，推动上思南屏等纳入边境地区乡镇建设规划，促进沿海和沿边开发开放协调发展。

（二）深入实施工业强市战略，构建现代版临港工业基地

以六大支柱产业为核心，六大园区为载体，改造提升现有产业，全力发展新兴产业，不断提升产业竞争力。

打造六大支柱产业。钢铁产业要以机械、船舶修造、汽车、集装箱等行业为重点，发展高附加值的精品钢材，延伸中下游配套产业。加快钢铁冷轧、热轧项目建设，确保上半年冷轧项目投产、热轧项目开工。开工建设盛弘机械等项目。有色金属产业要加快金川15万吨铜材深加工、盛隆二期建设，推动金源二期、艾好钼铼贵金属等项目开工，加快发展配套产业，形成有色金属产业集群。食品产业要发挥大海、岳泰、农垦糖业等大型食品企业的优势，做优做强油料加工、饲料加工和生态食品制造业。加快推进昌菱5万吨朗姆酒、汇圣40万吨啤酒项目建设。能源产业要发挥核电、中电等大型项目的带动引领作用，继续发展传统能源，大力发展绿色新能源。争取红沙核电一期一号机组上半年试产、二期年内开工建设，加快中电二期项目建设，推进三、四期前期工作，争取开工昌菱生物质发电、四方岭和南山风电、旭日升光伏电站和生态农业光伏产业园。石化产业要以信润、科元、中海油LNG等项目为重点，发展化工新材料产业。加快推进龙成项目，建设现代新兴清洁煤基地。以磷化产业园搬迁为契机，推动磷化产业二次创业。装备制造产业要以海森特、中一重工等项目为重点，发展海洋工程装备、特种钢构、港口机械等产业，建设北部湾海洋工程服务基地。以沙潭江科技工业区为载体，培育发展电子信息、生物技术、节能环保等高新技术产业，推动迪斯生物、钧富凰绿色空调等项目开工建设。

增强六大园区承载力。以六大产业园区为核心，加强基础设施建设，完善功能配套，提升产业集聚能力。企沙工业区要加快水、电、路、气、排污等基础设施建设，抓好赤沙南路、工业南路、云约江南路、玉石滩大道等主干道及污水处理厂建设。大西南临港工业园要加快建设榕木江大道、小龙门大街、污水处理厂等一批基础设施项目。东湾物流加工区要抓好排水系统、辅助路网、物流输送廊道等项目前期工作，加快220千伏航洋变电站等项目建设。跨境合作区要抓紧“一门一桥一厂一馆六路”建设，确保起步区基础设施竣工使用。沙潭江科技工业区要推进文昌大道东段建设，启动15万平方米标准厂房及配套设施建设。企沙东岛能源化工基地要做好项目规划策划，抓好跨海大桥及配套道路、码头、航道等基础设施建设前期工作，启动填海造地工程。

加快工业转型升级。抓好现有企业技改，推动盛隆冶金、大海粮油、惠禹粮油、中一重工等重点企业升级改造。突出创新驱动，以云朗科技园为平台，以电子信息、新能源、新材料等领域为主攻方向，培育壮大百亿元战略性新兴产业。发展循环经济，加快金川副产硫酸循环经济项目建设，深入推动昌菱、上上糖等企业循环经济发展。

（三）推动“十百千”项目建设，打造发展强大引擎

深刻认识经济发展新常态下抓重大项目的极端重要性，发挥投资的关键作用，抓好983个总投资6716亿元的项目建设计划，为全市经济社会发展提供强大支撑。

强力推进项目建设。按照“十百千”项目建设计划，继续推进十大龙头项目建设，加快建设钢铁基地、科元新材料、信润石化等项目，年内完成投资143亿元；抓好76个投资3亿元以上的重点项目，推进东盟农产品国际采购中心、四通八达国际广场等项目建设，年内完成投资135亿元；实施513个投资500万元以上的规上项目，年内完成投资222亿元。确保年内全市开工项目296个，续建180个，竣工投产174个。抓紧推进187个项目前期工作、完成146个招商项目的论证工作。

提高项目建设保障能力。突出问题导向，切实解决项目建设中存在的困难和问题，实施土地、资金、劳动力等生产要素倾斜政策，优先解决重大项目征地拆迁、用地指标、建设资金等问题。抓好低丘缓坡荒滩等未利用地开发利用试点，推进“旱改水”工作，做好土地收储，推进连片征地，加快“熟地”供应。妥善做好被征地群众搬迁安置工作，抓好住宅安置、产业安置、就业安置和失地失海农民社会保障。推动银企合作，加大融资力度，积极争取上级支持，千方百计落实项目建设资金。

实施项目精细化管理。落实我市《规上项目管理暂行办法》及《项目推进考评办法》等项目管理制度，按照“一个项目、一位领导、一套班子、一抓到底”的工作机制，强化跟踪督查和考核激励。加强和规范政府投资项目管理。严格实施项目招投标、合同管理、工程监理、竣工验收、预算决算审计等五项制度，简化审批，实行“同级审查”，形成部门协调联动、管理服务高效、组织实施规范的管理模式，保持项目“开工一批、在建一批、投产一批、储备一批”的滚动式发展态势。

（四）实施城市功能提升工程，加快建设生态宜居城市

以创建国家生态园林城市为目标，按照宜居、宜游、宜业、宜学的要求，投资158亿元，实施城建项目383个，不断完善城市功能，提升城市品质，使防城港市更有魅力。

强化城市功能配套。全面解决城市供水不足问题，三波水厂要在上半年、中心区配水厂要在下半年通水运营。保障城市燃气供给，中缅线公车接气站及配套管网要在上半年完成建设，降低气价。加快老城区管道天然气转换工作，确保城镇居民和企沙半岛大型企业用气需求。加快公车变10千伏出线、企沙变10千伏大龙线等电网项目建设，抓好城区电力架空线路入地和通讯光缆入地管道改造。加快城市污水垃圾处理项目建设，完善一批商场、市场、公园、广场、停车场、公厕等设施，为市民创造更加便捷的生活环境。

完善城市交通体系。加强城市路网建设，确保西湾环海大道、东湾大道、文昌大道等城市主干道年内竣工通车，加快建设江山半岛环岛路改扩建项目。推进常山新区、城南新区、江山大道以南等片区路网建设。抓好长途汽车客运站、公交车站、企沙客运站等交通项目建设。

打造城市核心景观带。落实创建国家园林城市计划，以百里黄金海岸景观带和边海大道为重点，实施一批城建重点项目。推进绿色慢道休闲空间建设，开工建设金花茶公园、沙潭江滨江公园、蜈蚣岭公园，启动西湾人文生态绿色廊道、边海大道绿化亮化提升工程，加快构建环西湾景观带。建设一批园林景观道路，年内建成桃花湖公园二期、东湾大道绿化工程等项目。做好园博园规划建设。抓好东兴城市风貌改造，统一沿线街景风貌，凸显东盟、边关、民族特色，打造魅力边城。推动上思“一江两岸”景观建设，打造生态山城。

推动产城融合发展。走新型城镇化道路，探索产城融合发展新模式，抓好企沙工业区、大西南工业区、云朗科技园及重大产业项目周边城镇发展，完善社区服务中心及文化、体育、教育、卫生等周边生活配套区设施建设，解决企业职工吃、住、行、娱乐、购物、就医、子女入学等实际问题。

提升城市管理服务能力。理顺城市管理体制，构建大城管格局。整合执法力量，提高城市综合管理执法水平。加强城市环卫、交通秩序、集贸市场的管理，整治泥头车，依法查处违法违规建设，规范管理门店招牌和户外广告。推进智慧城市建设，实施精细化、网格化管理，推广智能交通、智慧旅游、智慧照明等信息化应用。

（五）推进先行先试，增创开发开放新优势

充分发挥“两沿三区”优势，大胆改革，勇于创新，全面提升对外开放水平。

加大先行先试力度。推进企业登记制度改革、个人跨境贸易人民币结算等先行先试成果转化，深化行政审批、投融资体制、国资国企和北部湾经济区同城化、跨境经济合作区管理体制、贸易投资便利化等重点领域改革。抓好沿边金融、跨境金融、农村金融等探索创新。争取在境外边民劳务合作、互市区免税购物、跨境自驾游等方面实现新突破。

加快开放合作平台建设。以口岸经济为重点，打造中国—东盟门户城市。完善口岸基础设施，扩大港口口岸开放范围，推动峒中、里火等口岸升格，争取防城港口岸联检大楼上半年投入使用。拓展进口粮食指定口岸业务，提高东兴进境种苗（景观树）指定口岸运营效益，争取获批进境水果指定口岸。抓好综合保税区、防城边境经济合作区的前期工作。推进东兴边民互市贸易区、国际旅游集散中心、北仑河国际商贸城、峒中—横模口岸大桥建设，规划建设游客免税购物市场、金融一条街、北仑河二桥口岸区。

积极开展对外交流。采取“走出去、请进来”办法，开展经贸、文化、旅游、教育等多层次、宽领域的开放合作，形成参与国际合作的新格局和新优势。拓宽国际友城渠道，积极开展国际海上龙舟节、中越边境（东兴—芒街）商贸旅游博览会、京族哈节等活动，搭建对外交流平台。

（六）加强港航基础设施建设，打造国际枢纽大港

以建设5亿吨、500万标箱大港为目标，抓好50个总投资475亿元的港口项目建设，提高港航服务质量，确保完成港口货物吞吐量1.25亿吨，为建设国际枢纽大港打下更坚实的基础。

优化港口布局。加快《防城港港口总体规划》修编工作，推进茅岭、云约江、珍珠湾港点控制性规划编制。加快升级改造渔氵万港区，提高配套能力，拓展集装箱业务市场；企沙港区要围绕重大产业项目建设，做好大宗散货承接；江山、竹山等中小码头要围绕商贸物流和旅游开发，合理利用岸线资源。留足港口发展空间，加强港口粉尘治理，促进港口、城市、产业和谐发展。

实施一批港口码头重大项目建设。推进27个港口项目建设，力争完成投资17.8亿元。启动40万吨级散货码头和40万吨级航道建设，增强港口核心竞争力。抓好中电二期码头、401、501、502号泊位、潭油作业区3、4号泊位、第五作业区进港航道、潭油作业区进港航道和企沙南作业区航道一期等一批新项目建设。继续推进513-516号泊位、钢铁基地专用码头、信润石化码头和凌宇运通码头等一批续建项目。争取402号泊位、海事码头等一批重点项目竣工使用。

加快完善集疏运体系。抓好疏港公路、铁路、机场建设，打造区域性综合立体交通运输体系。开工建设企沙至茅岭一级公路、滨海公路东兴至江平段和钦防高速公路改扩建工程。抓好滨海公路龙门大桥西引道、防城至江山一级公路、马路至峒中公路等项目建设。启动东兴至凭祥沿边高等级公路、吴圩机场经上思至东兴高速公路的规划论证。加快南防铁路钦州至防城港段扩能改造工程、防城至东兴、茅岭至皇城坳等3条干线铁路和金川、钢铁、云约、潭油等4条支线铁路建设，抓好云约、皇城坳、茅岭等3个铁路枢纽站建设。做好支线机场规划研究和通用机场申报工作。

强化港航服务能力建设。推进航运中心建设，规划建设防城港航运交易所，引进船舶交易、船舶技术、航运经纪等各类航运服务行业，发展港口金融、保险、信息等港航服务机构，拓展港航服务产业链。加强通关能力建设，推行“三个一”通关模式，提高口岸服务水平，吸引更多货物从防城港通关。

（七）夯实“三农”基础，推动现代农业加快发展

深入实施农业产业化“336”工程，加快农业发展方式转变和农业产业结构调整，促进农民增收、农业增效、农村繁荣。

加快发展现代农业。继续保持粮食稳定生产，巩固东兴现代特色海洋渔业核心示范区，加快推进上思、港口、防城等现代特色农业核心示范区建设。大力发展设施农业，推进传统农业向高效农业转变。培育金花茶、中草药、坚果、油茶等特色产业，新发展“双高”糖料蔗基地1.5万亩、特色水果1万亩、水产健康生态养殖3万亩、肉牛养殖5万头。发展远洋捕捞业，抓好江山、江平、茅岭滩涂养殖区发展，推动北部湾鱼苗培育、深海网箱养殖基地建设。加强农产品生产和流通环节监管，确保农产品质量安全。

努力促进农民增收。深入实施“万元增收工程”，确保农民人均纯收入突破万元大关。扶持发展家庭农场、种植大户等新型经营主体，全年新建“万元片区”3万亩，“万元村屯”150个，“10万元示范农户”100户，带动全市优势特色农业发展，促进农民增收。深化农村改革，引导土地经营权有序流转，推进林权流转、林权抵押、森林保险等配套改革，抓好农村土地承包经营权确权登记试点，增加农民财产性收入。健全农业社会化服务体系，新发展农民专业合作社50家。抓好农民工转移就业培训，转移农村就业1.8万人，提高农民工资性收入。

加强生态乡村建设。巩固清洁乡村成果，抓好“美丽家园·生态乡村”建设，开展村屯绿化、饮水净化、道路硬化“三化”专项活动，以“一门两沿三江六线”为重点，实施村屯绿化1105个，打造生态乡村示范点200个，建设安全饮水工程100个，解决2.5万人农村安全饮水问题，完成100个以上村屯内主干道硬化，建设200公里以上通自然村屯道路。加强江河流域整治和海滩整治，治理水土流失面积30平方公里。加强农田水利建设，推进大垌水库等一批水利项目建设。完善乡镇垃圾中转站及公厕建设，建成企沙、江平、那良、在妙等重点镇污水处理厂。立足生态优势，着力转变发展方式，加快发展生态经济，培育生态林业、生态农业、有机食品加工业、生态健康养老业等新兴产业，着力打造生态文明示范区。

（八）大力发展第三产业，培育经济新的增长点

充分发挥大港口、大贸易优势，发展旅游、商贸、物流和现代服务业，确保第三产业实现增加值190亿元，增长6%。

推动旅游业大发展。以江山半岛、京族三岛、西湾新城、十万大山、大东沙等核心景区为重点，加快5A景区创建工作，抓好国际邮轮母港及航线项目，把旅游经济作为发展第三产业的龙头，加快打造现代服务业新高地。发展跨国旅游业务，用足用好边境旅游异地办证和东兴口岸落地签证政策，推进旅游通关便利化，争取开通中越跨境自驾游和陆海循环游。加快建设马鞍岭1、2号旅游码头，推进渭博·基斯顿等一批星级酒店建设，提升旅游接待能力。充分利用滨海、森林等丰富的生态资源优势，突出中国氧都、中国长寿之乡等品牌效应，开发滨海休闲度假、海上丝路、健康养生、养老保健等产品。发展生态旅游、乡村旅游，促进旅游产品特色化、品牌化，推进滨海休闲度假、十万大山健康养生、东兴长寿养生和防城港温泉疗养等四大健康养生基地建设。

推动商贸业大发展。实施“人气聚集工程”，推进城市中心区商圈建设。建成华美立家建材市场、中心区集贸市场，加快推进西湾集贸市场、金海海产品市场建设。推动电子商务体系建设，打造北部湾电子商务港。落实加工贸易倍增计划，推动边境贸易转型升级，支持边贸企业开拓国外市场。

推动物流业大发展。加快北部湾现代物流中心、国际汽车城、西南木材交易中心、东盟国际采购中心等项目建设。抓好传化公路港、东兴冷链物流加工中心、沙潭江国际采购中心等项目前期工作。加快北部湾商品交易所建设，争取北部湾矿产、粮油、木材等3家大宗商品交易市场落地。加快全市物流公共信息服务平台建设，提高物流业信息化水平。

推动生产性服务业大发展。围绕钢铁、能源、有色金属等支柱产业，引进和培育融资租赁、现代物流、检验检测、研发设计、中介咨询、信息技术服务、职业培训等一批市场潜力大、带动能力强、能够产生品牌示范效应的生产性服务企业，推动现代服务业优化升级。全面实施“抓大壮小扶微”工程，落实非公强优企业培育计划，推动非公经济发展。

推动互联网产业大发展。依托国家建设“中国—东盟信息港”机遇，争取海底光缆建设等通讯基础设施项目落户我市。建设移动电商运营基地，争取获得“跨境贸易电子商务支付牌照”资质，培育3-5家电子商务示范企业，筹备设立电子商务产业园区、创业基地，推进防城港“IT小镇”筹建工作，打造中国—东盟跨境电商交易中心和国家级电子商务示范基地。推动防城港“北斗数谷·数字广西”项目建设，启动建设北斗卫星大数据平台，发展推广北斗卫星终端服务应用，打造大数据产业。

推动金融业大发展。深入实施“引金入港”战略，抓好交通银行、兴业银行引进工作，争取更多保险公司在我市设立分支机构，新增3家小额贷款公司、1家融资性担保公司。推进北部湾产业基金和绿色环保健康产业基金运作管理，力争基金规模达13亿元。抓好港工公司12亿元债券发行、东投集团12亿元非公开定向融资工作。

（九）保障和改善民生，促进社会和谐发展

以教育、卫生、扶贫三大攻坚计划为重点，将更多财力向民生领域倾斜，促进社会事业蓬勃发展。

抓好教育攻坚计划。继续实施八大教育攻坚工程，加快建设市理工职业学校新校区、市高级中学二期、市五中、市六中；开工建设新实验高中；推进港口区大龙新区小学，防城区文昌小学、珍珠小学，上思县一中，东兴市华侨学校二期、北仑河中学二期等项目建设；加快城区及乡镇中心幼儿园建设。继续推进农村义务教育薄弱学校改造，创新教师队伍管理，加大培训力度，提升教学水平。

抓好卫生攻坚计划。继续开展五大卫生攻坚计划。加快城区医疗卫生设施建设，抓好市第一人民医院全科医生培训基地、住院综合楼、儿科楼和市中医院迁建等项目建设。不断完善乡镇、社区卫生服务中心布局，开工建设上思妇幼保健院综合楼和防城那梭镇、华石镇、茅岭乡中心卫生院业务用房。深入推进医改工作。创新人才培养使用机制，引进一批卫生人才，提高卫生队伍素质。

抓好扶贫攻坚计划。继续开展十万大山山区扶贫攻坚，以120个贫困村为重点，投入5.59亿元，实施160个项目，每个贫困村投入200万元以上。认真落实“一挂两包”制度，实施精准帮扶，逐村逐户分类制定帮扶计划，提高扶贫成效；以兴边富民行动大会战为重点，努力解决村屯道路、饮水、用电、沼气池、卫生、教育、文化、广播电视等问题，改善边民生产生活条件；抓好产业扶贫示范项目，培育扶贫龙头企业，助推更多农民增收项目落户。深入推进整市扶贫，确保年内脱贫1.2万人，贫困村农民人均纯收入增长12%，达6890元，20户以上自然村80%通沥青（水泥）路，基本解决群众饮水和住房安全问题。

抓好其它社会事业发展。加强科技创新，加快培育科技创新主体，强化知识产权保护，发展高新技术产业，提升科技服务信息化水平，推进企业主导的产学研合作。加快公共文化服务体系建设，推进全民健身运动。拓宽就业渠道，切实解决失海失地农民就业问题。加强社会保障，提高城乡居民养老金水平和最低生活保障标准，探索城乡老年居民、农民工等群体的养老保障办法，落实低收入群体帮扶救助措施，深入推进基本医疗保险城乡统筹，力争年内实现社会保险人群全覆盖。全力推进棚户区改造，完成改造6578户以上。强化食品药品安全监管，建立健全食品药品检验检测体系。加强生态文明建设，抓好节能减排和环境保护。继续推进我市率先基本实现气象现代化试点工作。加强社会组织培育管理，鼓励发展民间公益组织。完善应急预案体系，提高应对突发公共事件的能力。落实安全生产责任制，防范重特大安全生产事故发生。加强“六五”普法工作，推进依法治市。

加强国防动员和后备力量建设，开展国防教育、双拥共建活动，加强维权维稳力量及基础设施建设，深化军民融合式发展。强化社会治安综合治理，抓好边海防管控，加大打击走私力度，严厉打击各类违法犯罪活动，确保边海防和社会安全稳定。

实施十大民生工程。筹措资金10.74亿元，实施公共配套、交通便民、文化惠民、医卫利民、教育助学、保障性安居、水利惠民、生态建设、社保助民、食品安全等十大类重点民生工程，进一步改善群众生产生活条件。

（十）加强自身建设，努力打造法治政府

按照“职能科学、权责法定、执法严明、公开公正、廉洁高效、守法诚信”要求，推进依法行政，加快建设法治政府。

加强依法行政建设。遵循法治思维、运用法治方式行使各项权力，打造行政有为、运转高效的政府。完善重大问题集体决策、公众参与、专家咨询制度，认真听取各民主党派、工商联、无党派人士、人民团体和社会各界人士的意见。健全重大事项向人大报告制度，支持政协开展民主监督，对重大决策实行合法性审查、风险评估、跟踪问效。规范行政执法行为，加强执法监督检查，抓好重点领域专项治理。加强政务服务和公共资源交易监管体系建设，全面推进政务公开，加强政府债务管理，有序开展财政预决算和审计结果公开，主动接受社会和舆论监督。

加强执行能力建设。持续推进作风转变，强化政治意识，确保令行禁止。杜绝推诿扯皮、办事拖拉，推行一线工作法，以一抓到底抓落实的力度推动各项工作。加强干部培训，增强干部组织协调能力和化解复杂矛盾能力。强化重要决策部署的督查落实，严格行政问责，切实提高政府公信力和执行力。

加强廉政建设。全面落实中央八项规定，贯彻落实国务院“约法三章”，倡导勤俭节约，反对铺张浪费，严控“三公”经费支出。抓好部门权力清单制度建设，继续推进简政放权，确保上半年向社会公布市本级全部权力清单和责任清单，积极探索推行负面清单和财政专项资金管理清单。加大查处违法违纪案件力度，树立政府为民务实清廉的形象。

各位代表，“山高愈前行，梦好起宏图”。让我们紧密团结在以习近平同志为总书记的党中央周围，在市委的坚强领导下，坚定信心，改革创新，攻坚克难，狠抓落实，全力以赴完成全年任务和“十二五”规划目标，为争当全国沿边开发开放排头兵、加快迈入沿海开放城市第一集团而努力奋斗！
